# Consumption of young child formulae in the Netherlands

**DOI:** 10.1007/s00394-022-02956-2

**Published:** 2022-07-29

**Authors:** Marjolein H. de Jong, Eline L. Nawijn, Janneke Verkaik-Kloosterman

**Affiliations:** National Institute for Public Health and the Environment (RIVM), Bilthoven, The Netherlands

**Keywords:** Young child formulae, Micronutrients, The Netherlands, Habitual intakes

## Abstract

**Purpose:**

Adequate micronutrient intakes are essential for young children. Special young child formulae (YCF) intended for children from 1 year old are available in the Dutch market. Since YCF are enriched with many micronutrients, it has the potential to have a beneficial effect on young children, or might pose a risk on excessive micronutrient intakes. The current study investigated the characteristics of YCF users, and the effect of YCF use on micronutrient intakes.

**Methods:**

Data from the Dutch National Food Consumption Survey (2012–2016; *n* = 440 children aged 1–2 year old) and the Dutch Food Composition Database (NEVO version 2016) were used to assess micronutrient intakes. Habitual intakes of users and non-users of YCF were calculated using Statistical Program to Assess Dietary Exposure (SPADE) and compared.

**Results:**

In the Netherlands, YCF was consumed by 21% of the 1–2-year-olds. YCF contributed mostly to total vitamin D intake (76%) and between 0 and 50% for other micronutrients. Higher vitamin A, B1, C, D, E, total folate, iron and zinc intakes were observed among users, and higher potassium and phosphorus intakes were found among non-users. Risk of inadequate intake was low among both users and non-users for most nutrients, and the only elevated risk of excessive intake found was for zinc among YCF users.

**Conclusion:**

YCF increased micronutrient intake, however, for most of the micronutrients there is already a low risk of inadequate intake. YCF increased the risk of excessive zinc intake. It is important that the addition of micronutrients to YCF is regulated, to prevent excessive intake.

## Introduction

Adequate micronutrient (vitamins and mineral) intakes are essential for health [1]. Optimal nutrition is especially important for young children, as this is involved in healthy growth. Inadequate micronutrient intake may result in deficiency symptoms and eventually in deficiency diseases [2]. However, in addition to the risk of deficiency diseases, excessive micronutrient intakes can also be harmful and lead to adverse health effects on the long term [1].

A healthy diet can provide sufficient intake of micronutrients. This is also the case for young children, except for vitamin D and K, for which the Dutch government advises use of supplements [3]. For breastfed children, 150 µg vitamin K/day is advised in the first 12 weeks of life and for all children up to 4 years old 10 µg vitamin D/day is advised. To help parents providing micronutrients to young children, special formulas intended for children from 1 year old are available on the Dutch market. These so-called young child formulas (YCF) are marketed as the replacement of infant formula and follow-up formulas, when children are 1 year old. Similar to infant- and follow-up formulas, YCF are milk- or plant-based drinks fortified with many micronutrients. Although YCF are available, there is no Dutch recommendation to use this type of product [4]. Dutch parents are advised to stop with follow-up formula after the first birthday of the child and to offer regular (plant-based) dairy to their children.

Unlike infant formula and follow-up formula, there is no specific legislation for the composition of YCF [5, 6]. Since 2016, YCF falls under the legislation for regular foods [5], meaning it has to meet the fortification rules for regular foods. However, with the current composition of the YCF, which is generally based on follow-up formula, these rules cannot be met. In the Dutch legislation there are limitations on which micronutrients can be added and at what level. As YCF is such a specific type of food for a specific small population group, the Dutch government is working to produce guidance with minimum and maximum micronutrient contents for YCF [7].

Since YCF is enriched with many micronutrients, it has the potential to increase micronutrient intake and therefore might have a beneficial effect on young children. In the Dutch National Food Consumption Survey (DNFCS) 2012–2016, Van Rossum et al. (2020) did not find evidence of a low risk on inadequate vitamin D and iron intakes for young children [8]. On the other hand, the high micronutrient content might pose a risk on excessive intakes. Among Dutch young children, copper, zinc and vitamin A (retinol) intakes are high [8].

In this study, the contribution of YCF consumption on the micronutrient intake of children aged 1–2 year old was investigated. Characteristics of users of YCF were explored as well as the food pattern of (non-)users of YCF. Additionally, the contribution of YCF to the total micronutrient intakes was examined and the impact of YCF on the risk of inadequate or excessive intakes was investigated.

## Methods

### Survey population

In the Dutch National Food Consumption Survey (DNFCS) 2012–2016, data were collected on food consumption of the Dutch population (*N* = 4313, 1–79 yr.). A detailed description of the DNFCS 2012–2016 is described elsewhere [8].

The current study focused on children aged 1–2 year old. Food consumption of children was estimated by interviewing parents or caretakers about their child’s food consumption. For all children, two 24-h recalls on non-consecutive days were performed by trained dieticians using GloboDiet (IARC^©^; former EPIC-Soft). Height and weight were measured on the first recall day. Body mass index (BMI) was calculated per child as the body weight divided by the height squared (kg/m^2^) and categorized into seriously underweight and underweight, normal weight and overweight and obesity. Categorization was based on age-specific BMI cutoff values specified for gender and month of age [9]. With a general questionnaire, other general information of the participants was collected. Various background factors, such as educational level, native country, family size, and various general characteristics of the diet, such as special diets and eating habits were also recorded. The degree of urbanisation was divided in extremely urbanised (2500 or more addresses/km^2^), strongly (1500–2500 addresses/km^2^), moderately (1000–1500 addresses/km^2^), hardly (500–1000 addresses/km^2^) and not urbanised (fewer than 500 addresses/km^2^), as distinguished by Statistics Netherlands (CBS) [10]. The highest education qualification of the head of the household was recorded.

### Definition YCF

YCF were defined as milk- or plant-based drinks fortified with micronutrients and intended as the replacement of infant formula and follow-up formulas, designed for children aged 1 year and older. No distinction between milk-based YCF and plant-based YCF was made as there were only three children using plant-based YCF. Users of YCF were considered as user when they consumed at least one time a YCF product on at least one of the recall days.

### Food groups

Mean consumption in g/day from all food groups (according to the GloboDiet classification system [8]) was calculated for users and non-users of YCF separately. The GloboDiet classification consists of 18 main- and 78 subgroups. To study if non-users of YCF consume more regular milk than YCF users, also the mean intake from milk and breast milk was calculated. Milk included regular cow milk (skimmed, semi-skimmed and full fat), raw milk, buttermilk, milk enriched with calcium, semi-skimmed lactose free milk, full fat goat milk, almond milk, soymilk and rice milk. Breastmilk could only be quantified if the milk was pumped and then fed to the child. If the milk was not pumped, the quantity was assumed to be 70 ml for each feeding, based on the declining breast milk intake for older infants as assumed by the EFSA [11].

### Contribution of YCF to total micronutrient intake

Food consumption data from DNFCS 2012–2016 was linked to the Dutch Food Composition Database (NEVO; NEVO-online version 2016/5.0, RIVM, Bilthoven, 2016, with additions for the DNFCS 2012–2016) to estimate micronutrient intakes. Consumption data of YCF were linked to brand specific NEVO codes. To classify a formula as YCF, each NEVO code consumed within DNFCS 2012–2016 was checked to see it was YCF and not infant formula or follow-up formula. In this study we only considered the micronutrient intake from foods, intake from dietary supplements was excluded. The impact of YCF consumption was studied by calculating the contribution of YCF to total micronutrient intake. For these analyses, only the recall days on which a YCF was consumed were included. This means if a user consumed YCF on only one recall day, only that day was included.

### Assessment of risk on inadequate and excessive micronutrient intakes

The dietary reference values (DRV) adopted in 2015 by the Health Council of the Netherlands from the DRVs established by the Health Council of the Netherlands in 2000, 2003 and 2012, by the Nordic Council in 2012 and by the EFSA in 2006 were used in this study to assess the risk of inadequate and excessive intakes [1, 12–16]. An estimated average requirement (EAR) is a reference value sufficient for half of the population [17]. When scientific evidence is insufficient to establish an EAR, an adequate intake (AI) is proposed. This reference value is sufficient for almost everyone in the population. All the DRVs for adequate intake used in this study were AIs. For vitamin D, the AI for both sufficient as insufficient sunlight exposure was used for all children, as the amount of sunlight exposure for each child was unknown. To assess the risk on excessive intakes, the tolerable upper levels (UL) were used.

### Statistical analysis

To produce results representative of the Dutch population (in calendar year 2014) a weighting factor was applied in the analyses. This weighting factor included socio-demographic factors, as well as season and day of the week. As children were overrepresented in the study population (half of the study population consisted of children), the weight factor had a large impact on the population size after weighing. This weighted study population was used for the analysis of 1–2 year olds in current study.

General characteristics of YCF-users and non-users were compared with a Chi-Square test. The level of significance was corrected for multiple comparisons using the Bonferroni correction [18]. A *p* value < 0.007 was considered to indicate a statistically significant difference between the groups.

Mean consumption from food groups was calculated from the mean consumption of 2 recall days per participant for YCF users and non-users separately. The proportion of consumers of each food (sub)group was calculated as the number of users within food (sub)group divided by total subjects within the YCF non-user or user group. The percentage of difference in mean consumption within the food groups between the YCF non-users and users was assessed. Mean consumption from different food (sub)groups between users and non-users of YCF were compared using the Wilcoxon-Mann–Whitney test. The level of significance was corrected for multiple comparison using the Bonferroni correction and a *p* value < 0.0005 was considered as statistically significant.

Calculation of the contribution of YCF to total micronutrient intake and the habitual intakes was performed for the micronutrients calcium, copper, iron, magnesium, phosphorus, potassium, selenium, zinc, vitamin A (retinol, retinol activity equivalents (RAE) and beta-carotenes), vitamins B_1_, B_2_, B_3_, B_6_, B_12_, C, D and E, and folate (total folate (both naturally present and folic acid)), calcium, copper, iron, magnesium, phosphorus, potassium, selenium and zinc. For vitamin A expressed as RAE, 1 μg RAE was assumed to be equal to 1 μg retinol, 12 μg β-carotene and 24 μg other carotenoids [17]. Folate equivalents were calculated as the amount of folate naturally present in foods (in μg) and 1.7 times the amount of folic acid in enriched foods (in μg) [19].

For all micronutrients studied, the habitual intake (also referred to as usual intake) distribution was estimated by correcting the data for the within-person variation using the Statistical Program to Assess Dietary Exposure (SPADE version 4.0.85 of 16 December 2020) using R version 4.0.3. Intakes were age-dependently modelled for 1- and 2-year-olds, using the 1-part model.

The adequacy was only qualitatively assessed using an ‘adequate intake’ (AI), as no ‘estimated average requirement’ (EAR) was established for young children. A habitual median intake above the AI was considered as a low risk of inadequate micronutrient intake. If the median habitual intake was below the adequate intake, no statement about the adequacy could be made. If the 95% confidence interval (CI) of the median intake included the AI, we assumed a low risk of inadequacy. This assessment was made for 1- and 2-year-olds separately, based on the age-specific habitual intakes. Additionally, the proportion of the population with a habitual micronutrient intake above the UL was estimated. A proportion larger than 2.5% was considered high. Lower proportions were considered tolerable.

Habitual intakes and proportions above the upper level (UL) of users and non-users were compared by estimating the difference between users and non-users and calculating 95% CIs for these differences, based on 200 bootstrap iterations, similar to Dekkers and Slob [20]. If the 95% CI for difference did not include zero, habitual intakes or proportions above UL were considered as statistically different between users and non-users.

Statistical analyses were performed using SAS version 9 (Windows version 6.3.9600), unless stated otherwise.

## Results

### Characteristics of YCF users

Among the Dutch 1- and 2-year-olds, 21% were found to be users of YCF (Table 1). All the characteristics studied were comparable for users and non-users of YCF.

**Table 1:** Characteristics of YCF non-users and users for 1- and 2-year-olds^a^

	YCF non-users		YCF users		*P* value^c^
	Unweighted (*n* = 347)	Weighted (*n* = 75)^b^	Unweighted (*n* = 93)	Weighted (*n* = 2*0*)^b^	
Sex					0.1850
Boys	176 (51%)	38 (51%)	51 (55%)	12 (59%)	
Girls	171 (49%)	37 (49%)	42 (45%)	8 (41%)	
BMI					0.2157
(Extremely-) Underweight	32 (9%)	8 (11%)	4 (4%)	1 (5%)	
Normal weight	286 (83%)	61 (81%)	83 (19%)	17 (89%)	
Overweight/obesity	28 (8%)	7 (9%)	5 (5%)	1 (6%)	
Educational level^d^					0.3178
Low	14 (4%)	6 (8%)	3 (3%)	1 (5%)	
Middle	98 (22%)	27 (36%)	30 (32%)	9 (45%)	
High	235 (67%)	42 (56%)	60 (65%)	10 (51%)	
Urbanisation^e^					0.0398
Extremely/strongly	149 (43%)	36 (47%)	54 (58%)	12 (62%)	
Moderately	77 (22%)	15 (19%)	17 (18%)	3 (16%)	
Hardly/not	121 (35%)	25 (33%)	22 (24%)	4 (21%)	
Ethnicity					0.9145
Dutch	319 (92%)	68 (91%)	82 (88%)	18 (89%)	
Western immigrant	8 (2%)	2 (2%)	3 (3%)	0 (2%)	
Non-Western immigrant	20 (6%)	5 (7%)	8 (9%)	2 (8%)	
Season (First recall day)					0.2004
Spring	94 (27%)	21 (27%)	14 (15%)	3 (16%)	
Summer	71 (20%)	18 (24%)	23 (25%)	6 (30%)	
Autumn	80 (23%)	19 (25%)	23 (25%)	5 (26%)	
Winter	102 (29%)	18 (24%)	33 (35%)	5 (27%)	
Recall days					0.6288
Weekend/week	157 (45%)	36 (47%)	41 (44%)	9 (48%)	
Only week	125 (36%)	26 (35%)	31 (33%)	6 (31%)	
Only weekend	65 (19%)	13 (18%)	21 (23%)	4 (21%)	

^a^Weighted for socio-demographic factors, season and day of the week. As children were overrepresented in the original sample, the weight factor had a large impact on the study population size

^b^As a result of the use of a weight factor, results needed to be rounded to numbers without decimals, resulting into some groups of non-users and/or users with an n not equal to 75 and/or 20

^c^*P* value calculated with the Chi-square. *P* < 0.007 was considered statistical significant after the Bonferroni correction

^d^Highest educational level of the parents

^e^Extremely/strongly urbanised: > 1500 addresses/km^2^, moderately urbanised: 1000–1500 addresses/km^2^, hardly/not urbanised: < 1000 addresses/km^2^ [10]

### Consumption within food (sub)groups

YCF users had a 25% significantly lower mean consumption of the food group non-alcoholic beverages among YCF users compared to non-users. Besides, YCF users seemed to have a 32% higher dairy consumption compared to YCF non-users from the subgroup ‘non-fermented milk and milk beverages’, which included YCF products as well as regular milk (Table 2). Among YCF users there were fewer consumers of regular milk (cow/goat/plant based), compared to YCF non-users (30%-point lower). Additionally, the regular milk consumers among the YCF users consumed on average 23% less compared to the regular milk consumers among YCF non-users YCF.

**Table 2:** Mean consumption (in grams) of foods within food (sub-)groups of YCF non-users and users aged 1–2 years old

Food (sub-)group^a^	YCF non-users		YCF users		% of difference of mean consumption (%)^d^	*p* value^e^
	Proportion of consumers (%)^b^	Mean consumption (g/day)^c^	Proportion of consumers (%)^b^	Mean consumption (g/day)^c^		
Potatoes	79	68	79	62	− 8	0.389
Unclassified, mixed and other tubers	2	43	5	27	− 37	0.075
Potatoes	79		79	63	− 8	0.475
Vegetables	94	68	95	59	− 13	0.201
Unclassified, mixed salad/vegetables	18	36	22	30	− 16	0.214
Leafy vegetables (except cabbages)	24	44	17	28	− 35	0.381
Fruiting vegetables	64	51	69	45	− 12	0.649
Root vegetables	31	47	38	40	− 16	0.362
Cabbages	31	61	32	44	− 28	0.084
Mushrooms	13	13	13	10	− 28	0.991
Grain and pod vegetables	11	31	16	17	− 45	0.086
Leek, onion, garlic	40	11	52	10	− 9	0.362
Stalk vegetables, sprouts	7	10	9	16	56	0.524
Legumes	7	59	13	59	0	0.987
Fruits, nuts, seeds and olives	98	162	100	164	1	0.994
Fruits	94	151	91	148	− 2	0.570
Fruit compote	27	94	40	106	13	0.146
Nuts, peanuts, seeds	5	14	5	5	− 63	0.052
Peanut butter, nut/seeds spread	29	16	23	17	5	0.604
Olives	2	13	2	18	37	0.699
Dairy	100	400	100	476	19	0.055
Unclassified and mixed dairy products	5	120	1	96	− 20	0.523
Non fermented milk and milk beverages	88	272	99	359	32	0.002
Milk (cow/goat/plant-based)^f^	88	256	59	196	− 23	0.012
Breastmilk^f^	4	338	5	164	− 52	0.371
Fermented milk, milk beverages and yoghurt	36	216	25	210	− 3	0.922
Milk substitutes and milk substitute products	5	188	6	183	− 3	0.764
Yoghurt	40	121	31	104	− 14	0.158
Fromage blanc, petits suisses	23	81	29	69	− 14	0.152
Cheeses (including spread cheeses)	70	23	68	21	− 7	0.468
Cream desserts, puddings (milk based)	43	150	26	163	8	0.175
Unclassified creams	0	33	1	5	− 84	0.540
Dairy creams and creamers	11	13	10	7	− 49	0.592
Ice cream (milk based)	10	42	9	24	− 41	0.193
Sorbet/water ice	12	54	8	25	− 54	0.001
Cereals	100	102	100	101	− 2	0.554
Flours, starches, flakes, semolina	23	16	30	13	− 16	0.698
Pasta, rice, other grains	50	54	61	54	0	0.887
Bread	99	75	98	70	− 7	0.376
Crispbread, rusks	34	10	29	11	15	0.338
Breakfast cereals	30	29	45	24	− 19	0.004
Dough and pastry	6	50	3	29	− 43	0.386
Meat	97	45	94	38	− 17	0.057
Unclassified and combined meat and meat products	3	35	2	54	56	0.561
Unclassified, mixed and other mammals	9	32	9	17	− 48	0.128
Beef	20	28	22	19	− 33	0.339
Pork	17	36	12	26	− 29	0.332
Chicken, hen	28	30	36	29	− 6	0.282
Turkey, young turkey	2	23	7	20	− 15	0.337
Hot processed meat	56	49	37	49	− 1	0.925
Cold processed meat	68	20	55	21	4	0.581
Hot meat substitutes	2	30	2	19	− 37	0.556
Cold meat substitutes	2	18	3	23	30	0.897
Fish	17	54	15	56	3	0.837
Fish	8	47	9	57	22	0.948
Crustaceans, molluscs	2	34	2	50	47	0.817
Fish products, fish in crumbs	9	61	5	52	− 14	0.749
Eggs	34	31	20	26	− 17	0.457
Fat and oils	100	12	100	11	− 8	0.381
Unclassified and combined fats	17	4	15	2	− 50	0.048
Vegetable oils	50	3	67	4	11	0.561
Butter	16	7	17	6	− 10	0.628
Margarines and cooking fats	92	11	84	10	− 5	0.638
Other animal fats (including fish oils)	0	2	2	2	− 8	1
Sugar and sweets	90	20	82	18	− 13	0.362
Sugar	22	5	12	2	− 55	0.005
Jam, jelly, marmelade	21	21	30	15	− 27	0.911
Honey	7	6	4	2	− 68	0.205
Other sweet spread	28	7	29	7	− 5	0.614
Sweet sauce, sweet topping for desserts	1	4	0	0	n/a	
Syrup (incl. from can and for beverages)	5	12	7	5	− 61	0.189
Unclassified and other chocolate confectionery	28	10	17	12	25	0.808
Chocolate tablet	13	12	5	15	24	0.185
Chocolate candy bars	2	26	2	21	− 19	0.868
Chocolate spread and chocolate powder	24	22	20	24	5	0.403
Chocolate confectionery	7	13	0	0	n/a	
Confectionery non chocolate	44	12	30	13	1	0.241
Cakes and biscuits	82	28	82	23	− 17	0.134
Cakes, pies, pastries, puddings (non milk)	32	38	25	32	− 18	0.177
Dry cakes, sweet biscuits	75	20	77	18	− 9	0.178
Non-alcoholic beverages	100	590	100	443	− 25	**0.0003**
Fruit and vegetable juices	329	190	30	149	33	0.138
Carbonated/soft/isotonic drinks, diluted	88	454	80	364	81	0.002
Tea	21	183	17	126	19	0.035
Herbal tea	11	219	7	82	10	0.007
Waters	74	177	84	170	86	0.882
Condiments	65	17	57	16	− 2	0.424
Unclassified or combined condiments and sauces	1	15	2	35	137	0.136
Other and mixed sauces	34	20	13	20	− 1	0.874
Tomato sauces	16	18	20	23	26	0.467
Dressing sauces, mayonnaises and similar	22	13	12	15	16	0.732
Mayonnaise-based spreads	3	21	3	16	− 20	0.267
Spices, herbs and flavourings	1	0	1	0	− 49	0.540
Unclassified and combined condiments	21	3	28	3	11	0.172
Vinegar	1	1	2	3	98	1
Soups and stocks	13	112	11	86	− 23	0.661
Soups	6	112	2	79	− 29	0.956
Stocks	11	72	11	70	− 3	0.606
Savoury snacks	46	21	47	30	42	0.397
Unclassified or combined snacks	0	11	0		n/a	
Savoury snacks, biscuits and crisps	43	15	39	19	32	0.759
Savoury filled buns, croissants	8	55	9	67	20	0.832

Bold that indicates the non-alcoholic beverages with *p*-value 0.0003

^a^Foods categorised according to the GloboDiet food groups classification system

^b^Calculated as proportion of YCF (non-)users who consumed a food within the food (sub-)group on at least 1 of the recall days

^c^Mean consumption of users of the specific (sub-)group, weighted for socio-demographic factors, season and day of the week

^d^Difference calculated as the mean consumption of YCF users divided by the mean consumption of non-YCF users *100%

^e^*P* value calculated with the Wilcoxon–Mann–Whitney test. *P* < 0.0005 was considered statistical significant after the Bonferroni correction. Bold indicates a statistically significant different mean consumption

^f^No GloboDiet-subgroup

For most food groups, the proportion of consumers within each food group was equal among the YCF users and YCF non-users (Table 2). A higher proportion of consumers within the YCF user group (≥ 5%-point) were observed for the legumes group, and a lower proportion (≤ 5%-point) for the meat, eggs, sugar and sweets, and condiments groups compared to non-users of YCF. Within the subgroups, more variation of the proportion consumers between the YCF users and non-users was observed. The proportion of consumers was especially higher among YCF users for breakfast cereals and vegetables oils and lower for other- and mixed sauces and hot-and cold processed meats, compared to non-users of YCF.

### Contribution of YCF to total micronutrient intake

**Fig. 1:**
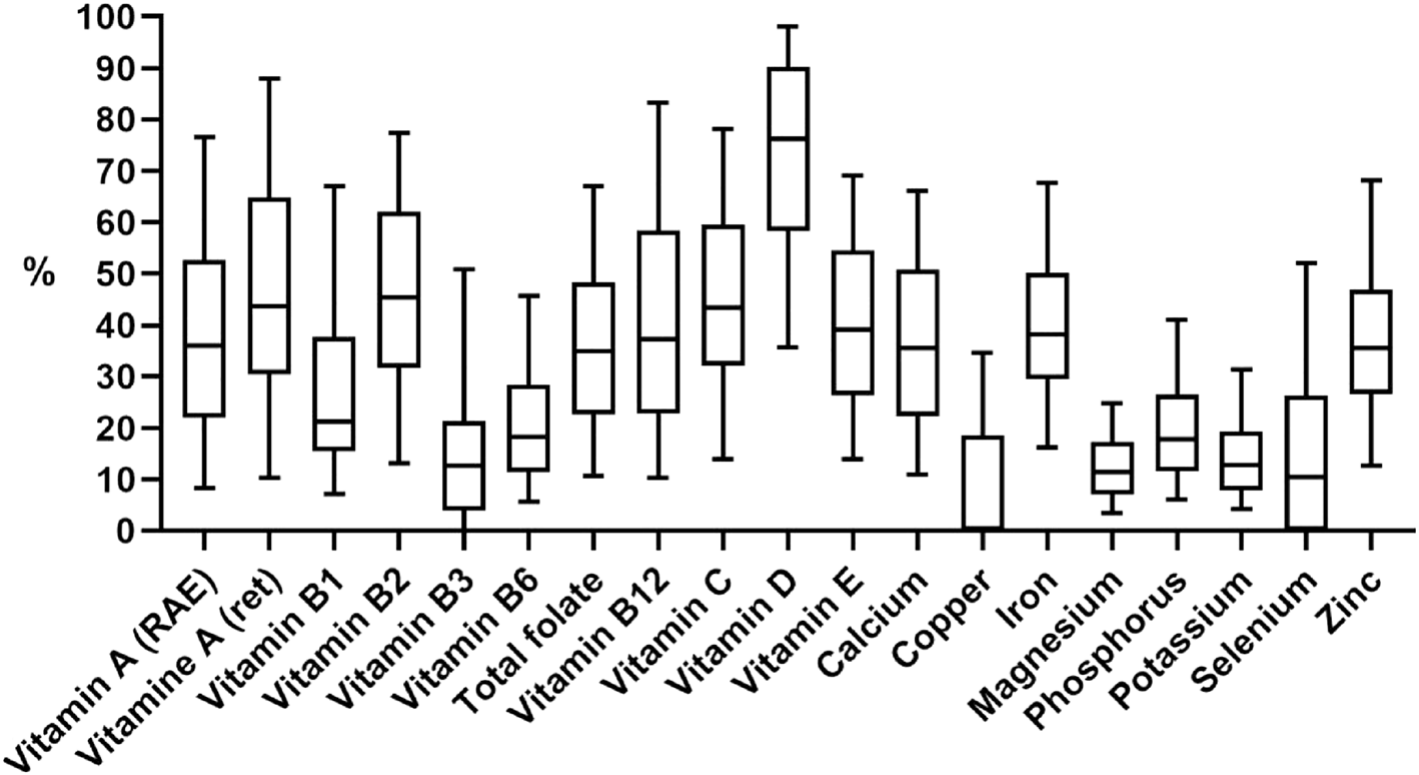
Contribution of YCF to the total micronutrient intake of the users (1–2 yrs old) of YCF. Upper whisk: P95, upper part boxplot: P75, middle line boxplot: P50, lower part boxplot: P25, lower whisk: P5

The intake of YCF contributed to the total intake of all micronutrients (Fig. 1). The median contribution to total micronutrient intake among users of YCF was the highest for vitamin D (median: 76%). For most of the other micronutrients, the median contribution to the total intake varied between 0 and 50%. The 0% value being for copper, as the contribution of other foods to total copper is higher. The variation in contribution among subjects was the largest for vitamin A (retinol) and vitamin B12, ranging from 10 to almost 90% (P5-P95).

### Adherence to the dietary reference values of users and non-users of YCF

For most micronutrients, the median habitual intake of users as well as non-users of YCF was above the adequate intake (AI), indicating a low risk of inadequate micronutrient intakes in the Dutch 1–2 year olds (Table 3). For YCF non-users and users, the 95% CI of the median potassium (2-year-olds) and selenium (1-year-olds) intake included the AI. For these nutrients, a low risk on inadequate intakes is likely. For all children, the median habitual intake of vitamin D was below the AI. Therefore, no statement was possible on the risk of inadequate intakes and additional research is required to study the (in)adequacy.

**Table 3:** Median habitual intakes of children aged 1- or 2-year-old YCF non-users and users compared to the adequate intake (AI) and upper level (UL)

			YCF non-users			YCF users		
Micronutrient	AI^a^	UL	Median habitual intake (95% CI)^b,c^	Evaluation risk inadequate intake^d^	% > UL (95% CI)^c^	Median habitual intake (95% CI)^b,c^	Evaluation risk inadequate intake^d^	% > UL (95% CI)^c^
Vitamin A (µg RAE)	300/350	–	492 (448–538)	LR	–	570 (492–650)	LR	–
Vitamin A (µg retinol)	–	800	379 (339–419)	–	8.9 (3.6–13.8)	448 (382–515)	–	11.4 (1.4–20.1)
Vitamin A (µg beta-carotene)	–	–	1091 (882–1296)	–	–	1078 (725–1467)	–	–
Vitamin B1 (mg)	0.3	–	0.6 (0.6–0.6)	LR	–	**0.7 (0.6–0.7)**	LR	–
Vitamin B2 (mg)	0.5	–	1.0 (0.9–1.0)	LR	–	**1.1 (1.0–1.2)**	LR	–
Vitamin B3 (mg)	4	–	7.9 (7.6–8.2)	LR	–	7.9 (7.2–8.6)	LR	–
Vitamin B6 (mg)	0.4	5	0.9 (0.9–1.0)	LR	0 (0–0)	0.9 (0.9–1.0)	LR	0 (0–0)
Total folate (µg)	85	–	131 (126–137)	LR	–	**162 (151–174)**	LR	–
Folic acid (µg)	–	200	2.3 (0.6–4.3)	–	0 (0–0)	**39.1 (33.8–44.2)**	-	0 (0–0)
Vitamin B12 (µg)	0.7	–	2.4 (2.3–2.6)	LR	–	2.2 (1.9–2.4)	LR	–
Vitamin C (mg)	25/30	–	63 (59–67)	LR	–	**81 (74–88)**	LR	–
Vitamin D (µg)	10/3^e^	50	2.0 (1.8–2.1)	NSP/NSP	0 (0–0)	**7.5 (6.5–8.4)**	NSP / LR	0 (0–0)
Vitamin E (mg)	4/5	100	6.4 (6.1–6.7)	LR	0 (0–0)	**7.7 (7.0–8.3)**	LR	0 (0–0)
Calcium (mg)	500	–	671 (644–699)	LR		667 (622–708)	LR	–
Copper (mg)	0.3/0.4	1	0.7 (0.7–0.7)	LR	1.7 (0.2–3.5)	0.7 (0.7–0.8)	LR	5.1 (0–10.1)
Iron (mg)	8	–	5.2 (4.9–5.4)	NSP	–	**8.0 (7.5–8.5)**	CIAI	–
Magnesium (mg)	85/120	–	174 (169–180)	LR	–	164 (154–174)	LR	–
Potassium (mg)	1400/1800	–	**1806 (1751–1862)**	1 year: LR 2 year: CIAI	–	1670 (1572–1772)	1 year: LR 2 year: CIAI	–
Phosphorus (mg)	470	–	**824 (798–851)**	LR	–	741 (694–786)	LR	–
Selenium (µg)	20/25	60	21 (20–22)	1 year: CIAI 2 year: NSP	0 (0–0)	23 (20–27)	CIAI	0 (0–0.2)
Zinc (mg)	5/6	7	5.3 (5.1–5.4)	1 year: CIAI 2 year: NSP	8.7 (4.6–12.7)	**6.2 (5.8–6.5)**	1 year: LR 2 year: CIAI	**27.9** **(15.3–38.5)**

*LR* low risk, *NSP* no statement possible, *CIAI* 95% confidence interval of median intake includes AI

^a^For vitamin A, C, E, copper, magnesium, potassium, selenium and zinc different AI values for the 1- and for the 2-year-olds were established

^b^Median habitual intake of 1- and 2-year-olds

^c^Significant higher median habitual intakes or higher proportions above the UL between non-users and users are displayed as bold

^d^Evaluation of risk on inadequate intakes separately performed for 1- and 2-year-olds

^e^Two AI-values for vitamin D, where 3 µg/day indicates adequate vitamin D intake with enough sun exposure and 10 µg/day if this amount of sun exposure is not met

Higher median habitual intakes were observed for YCF users compared to non-users for vitamin B1, B2, C, D, E, total folate, iron and zinc (Table 3). Of these nutrients, the higher intake of vitamin D (when the assumption of sufficient sunlight exposure is made), iron and zinc (2 year olds) resulted in an AI within or above the 95% CI of the median habitual intake. AI remained below 95% CI of the median intake for non-users. The higher micronutrient intakes of users resulted in higher percentages of children with intakes above the UL for zinc for users (28%) versus non-users (9%).

Lower median habitual intakes were observed for YCF users compared to non-users for potassium and phosphorus (Table 3). These lower intakes did not necessitate a different statement on the risk on inadequate intakes.

## Discussion

About one fifth of the Dutch children aged 1- and 2 years old could be considered to be a YCF user. YCF contributed to the daily intake of all micronutrients studied. In general, for most micronutrients (vitamin A, B1, B2, B3, B6, B12, C, E, total folate, calcium, copper, magnesium, potassium, phosphorus, selenium (1-year-olds) and zinc (1-year-olds)) young children (users of YCF as well as non-users) had a low risk of inadequate intakes.

The higher micronutrient intakes among YCF users only resulted in median intakes above or equal to the AI for vitamin D (and then only if sufficient sunlight exposure was assumed), iron, selenium (2-year-olds) and zinc (2-year-olds). No equivalent statement was possible for the non-users of YCF. For most of the other micronutrients for which higher intakes were observed among users, the non-users already had a low risk of inadequate micronutrient intakes and therefore a higher intake did not change these conclusions.

As there were fewer consumers of regular milk and non-alcoholic beverages among users of YCF and these consumers had a lower intake of these foods, YCF was probably used as a substitute for regular milk and other drinks. Cow-, goat-, or plant-based milks are rich in micronutrients just like YCF. However the micronutrient composition differs slightly. According to the Dutch Food Composition Database, cow-, goat-, or plant-based milk do not contain copper, iron, vitamin D and E, while YCF does [19]. Additionally, the selenium, zinc, vitamin A and C-content of YCF is higher than regular milk. This may at least partly explain the higher habitual vitamin C, D, E, iron and zinc-intakes among users of YCF. On the other hand, cow-, goat-, or plant-based milks have a higher calcium, potassium, magnesium, phosphorus, and beta-carotene-content, possibly explaining why non-users had higher habitual potassium and phosphorus intakes. When we replaced the YCF intakes of YCF users with cow milk, habitual iron, vitamin A, B1, B3, C, D, E, total folate, copper, selenium and zinc intakes decreased and calcium, vitamin B12, potassium and phosphorus intakes increased. This, however, only resulted in additional median intakes below the AI for iron, selenium (2-year-olds) and vitamin D (when sufficient sunlight was assumed) for YCF users. For all other micronutrients the conclusions when comparing to the AI remained the same as when YCF was consumed. These findings suggest milk consumed as a substitute for YCF results into the same conclusions for most of the micronutrients when comparing to dietary reference values as when YCF was consumed.

Our study showed that the median contribution of YCF to total iron intake was almost 40%. Iron intakes were higher among YCF users compared to non-users and resulted in low risk of inadequate intakes for 1-year-olds. Therefore, YCF has the potential to positively impact iron intakes of young children. A recent study by Sacri et al*.* (2021) support these findings as French children with higher serum ferritine levels are more often YCF users [21]. In an intervention study funded by the YCF-industry, Akkermans et al*.* (2017) showed that among Dutch, German and English children aged 12–36 months receiving YCF, baseline serum ferritine levels did not change after 20 weeks, while those receiving a non-fortified alternative had lower baseline levels [22].

Our study also found vitamin D-intakes were higher among YCF users, however median intakes were only above the AI for vitamin D for YCF users when sufficient sunlight exposure was assumed. This study does not conclude if sufficient sunlight can be assumed for Dutch children. Vitamin D levels raised among YCF users in the trial by Akkermans et al*.* (2017), decreasing vitamin D deficiency to 14%, while this increased in the control group to 33% [22]. For children in the Netherlands, a vitamin D supplement of 10 µg/day is advised [23]. 62% of the children aged 1–2-year old within the DNFCS 2012–2016 adhered to this advice on at least one of the recall days. Among YCF users this proportion was slightly higher, with 70% compared to 60% among the non-users of YCF. An increase in the proportion of young children achieving the levels advised on all days can result in median habitual intakes above the AI and therefore a low risk on inadequate intakes within the population.

Since 2016, it is clear that YCF does not fall under EU legislation for specific groups, but should be considered as regular fortified food [5, 6]. According to the Dutch government, fortified foods including YCF are not necessary for young children when the food patterns of children are divers and adequate [7]. When there is a risk of insufficient intakes, fortified foods, including YCF, could play a role. The Dutch government therefore intends to create specific legislation for YCF, including maximum micronutrient amounts, but this has not yet been published. This study showed YCF users had a larger risk of excessive intakes for zinc. The proportion above the UL among YCF users was three times higher compared to the non-users. Of the young children within the DNFCS 2012–2016, 15% consumed a zinc supplement on one of the recall days, and among YCF this was 10%. From the DNFCS 2012–2016, it was shown zinc intakes from supplements contributes to on average 4% of the total zinc intake of children aged 1–3 year old. In current study, nearly 30% of the 1–2-year-olds have intakes above the UL. Supplement intake was not correlated with high zinc intakes from food, therefore this proportion may be even higher when supplements are also considered. For the other nutrients, dietary supplements contributed 0–8% to the total micronutrient intakes. Therefore, no changes in the conclusions on the risk of inadequate or excessive intakes in this population group were expected from when only intake from foods were considered.

Dietary reference values are generally set for age-groups rather than age-years. For children, the values are often extrapolated from values set for adults based on body weight or body surface. This study only considered 1–2-year-old children, as they are the main consumers of YCF. We compared the intakes of 1-and 2-year-olds with DRVs for larger age groups, i.e., 1–3-year-olds or 2–5-year-olds. Body weight differences are large among these younger age categories, possibly resulting in incorrect statements on inadequacy or excessive intakes. As a result, the AI is probably too high for 1-year-olds and correct for 2-year-olds. However, as we compared two groups within the same age categories, the reported difference is expected to be accurate. For example if an AI is set for 1–3-year-olds, generally a body weight of 2-year-olds is used for the extrapolation. If a group consisting of 1–3-year-olds is compared with this DRV, the overestimation of inadequacy among 1-year-olds is corrected with an underestimation among 3-year-olds. However, in our study we had only 1–2-year-olds consuming YCF. Besides, the AI is a less strong DRV and has uncertainty. It is recommended to study the effect of correcting the DRV for the age-category for which the intake is studied, to see the effects of this problem.

As there were only three children using plant-based YCF in this study, we combined users of milk-based and plant-based YCF in the YCF user category. The micronutrient content of plant-based YCF differs from milk-based YCF, having some lower but also some higher content of specific micronutrients [19]. Although this difference in micronutrient content might change the risk on inadequate micronutrient intakes, also more information is needed on the dietary pattern of these children, as this may also differ between the milk-based and plant-based YCF users. Due to the low number of children consuming plant-based YCF, we could not study if there were any differences in dietary patterns between the two groups. Future research should study differences in dietary patterns and the risk on inadequate micronutrient intakes in populations with larger amount of children consuming plant-based YCF.

This study was based on data from the DNFCS 2012–2016. Data from the DNFCS 2012–2016 are representative for the Dutch population and with the extensive questionnaire and interviews for dietary assessment, the intake of Dutch 1-and-2-year-olds can be accurately estimated. The food consumption information was linked to NEVO-database, a comprehensive database which includes most of the foods consumed within the Dutch population. The coverage of the micronutrient content of this database is high, as it is between 71% and 99%. Micronutrient content was assumed to be zero if micronutrient content was not known, possibly resulting into underestimation of the true content. Food consumption data collected over two recall days is not representative for usual food consumption, as consumption may vary from day to day. With SPADE we could correct for these so-called within-person variances, resulting in habitual intakes of the population. These habitual intakes could be used to evaluate the adherence to the dietary reference values.

## Conclusion

One-fifth of the young children in the Netherlands use YCF. Although YCF increases micronutrient intake, for most of the micronutrients a low risk of inadequate intakes exists among users as well as non-users of YCF. For most micronutrients the additional intake from fortified YCF seems unnecessary to achieve adequate intakes. The consumption of YCF increased the risk of excessive zinc intakes. It is important that the addition of micronutrients to YCF is regulated, to prevent excessive intakes.

## Data Availability

Data of the DNFCS 2012–2016 are available on request from https://www.rivm.nl/en/dutch-national-food-consumption-survey/data-on-request (Accessed on 20 May 2021).
